# Recover aged/damaged erythrocytes and prevent them from aging by remodeling or enhancing their vital proteins: Hb and B3p

**DOI:** 10.3389/fcell.2026.1781681

**Published:** 2026-04-29

**Authors:** Guang-Yun Jiang, Wei-Wei Tuo, Zhuo Wang, Xing-Yao Chen, Yao-Xiong Huang

**Affiliations:** Department of Biomedical engineering, Jinan University, Guang Zhou, China

**Keywords:** mammalian erythrocytes, hemoglobin and band 3 protein, aging and disease, rejuvenation, flavonoid

## Abstract

This study investigates whether remodeling/enhancing the two vital proteins—hemoglobin and B3p—in erythrocytes with artificial interference can rejuvenate aged and damaged erythrocytes. Using Raman spectroscopy and SDS measurement, the study demonstrated that certain flavonoids, such as Hydroxysafflor Yellow A, could remodel/enhance the two proteins and restore their conformation, content, and distribution in aged erythrocytes within a few hours. As a result, it led to a restoration of nearly all cellular and molecular properties of old erythrocytes aged 90–120 days and cells stored under blood bank conditions for 21 days. The flavonoids could even recover erythrocytes of α/β-thalassemia. Moreover, pre-incubating the cells with flavonoids can prevent aging and damage caused by mechanical injury, hypoxic stress, acid-base imbalances, and storage lesions, with protective effects lasting for at least 21 days. In summary, this research reveals for the first time that remodeling hemoglobin and B3p with flavonoids is a promising intervention to enhance the longevity and resilience of erythrocytes against damage. It holds significant implications for biomedicine, including blood storage, the production of blood products, disease treatment, and high-altitude medicine related to hypoxic physiology.

## Introduction

Human erythrocytes, also known as red blood cells (RBCs), are the most abundant cells in the human body. They are vital in sustaining life by delivering oxygen to tissues and organs while removing carbon dioxide waste. However, their lifespan is limited to just 120 days. Throughout their circulation in the body, erythrocytes endure constant mechanical and oxidative stress. As a result of these stresses, they become aged and senescent before eventually reaching the end of their life cycle.

During their aging process, erythrocytes undergo a series of changes in structure and functions, as well as a reduction in mass and energy and a decline in physical and chemical properties. Erythrocytes do not have nuclei or other cellular organelles, so they do not contain DNA or RNA and cannot divide or repair themselves. They can neither produce new structures nor repair proteins or enzymes. Consequently, their components decrease with time.

Hemoglobin accounts for about 96% of the red blood cells' dry content and about 1/3 of the total content. They are the prime target of oxidative attack during circulation. Upon oxidation, oxy-Hb would auto-oxidize to metHb (Umbreit, 2007) and then aggregate or attach to the inner surface of the cell membrane (Huang et al., 2011a; Kang et al., 2008) at the cytoplasmic domain of band three proteins (Demehin et al., 2002). The conversion of Hb from oxy-Hb to metHb reduces the oxygen binding capacity of Hbs. The aggregation of Hb molecules and their attachment to the cell membrane, on the other hand, leads to an increase of Hb solution viscosity and a decrease in spectrin activity (Antonelou et al., 2018) so that the cell deformability decreases and fragility increases. There are also about 20% of the intracellular Hb proteins lost from the cell by vesiculation (Willekens et al., 1997; Willekens et al., 2003). Such a significant loss of intracellular proteins would reduce the red blood cell volume and affect the cell’s survival.

Band 3 protein (B3p), the most abundant RBC membrane protein and the main link between the cytoskeleton and the lipid bilayer of the cell, is also impacted by oxidative stress. The intense Tyr phosphorylation induced by oxidation at the cytoplasmic domain of B3p proteins would reduce their binding to anchor proteins, increase their lateral mobility, and promote the dissociation of B3ps from anchor proteins, thus causing their aggregation (Yang et al., 2024). This results in significant damage to the stability of the skeleton network and the reduction in the deformability of erythrocytes.

At the same time, there is a massive substance change of sialic acid (SA) molecules from the cells’ surface due to the constant mechanical and oxidative stresses during circulation. The old RBCs aged from 90 to 120 days only bear 70%–80% SAs as that of young cells. The cells stored in blood bank condition for 21 days also lose 47% of their SAs. SAs govern many cellular and molecular behaviors of erythrocytes, including morphology, membrane deformability, cellular adhesiveness, structure, distribution, and the oxygenation capacity of intracellular hemoglobin molecules (Durocher et al., 1975; Huang et al., 2016; Huang et al., 2011a). The loss of its surface sialic acid leads to a 20%–30% decrease in the cell’s surface charges/zeta potential (Danon and Marikovsky, 1961; Huang et al., 2011a; Huang et al., 2011b; Tuo et al., 2014; Yaari, 1969). Therefore, the old cel1s tend to aggregate with each other to form rouleaux or rosettes and adhere to endothelial cel1s (Baskurt and Meiselman, 2003; Ermolinskiy et al., 2020; Tuo et al., 2014) due to fewer electrical repulsion forces between them. It would also induce a series of topological structural changes in the membrane, including the exposure of D-galactosyl residues (Dhermy et al., 1987), the decrease in the ability of mucin to protect against oxidants, etc.

Hbs, B3ps, and SAs are the most abundant components, respectively, inside the cell, on the cell membrane, and on the outermost surface of RBCs. They also play significant roles in keeping the cell’s structure and functions and in the interactions of erythrocytes with environmental factors and other cell types. Therefore, the decline of Hb, B3p, and SA, can be considered the pivotal factors and events of erythrocyte aging (Huang, 2026).

As aforementioned, mammalian red blood cells lack nuclei to divide or repair themselves. They can neither produce new structures nor repair their proteins and enzymes themselves. However, one may complement or strengthen the decline components with some artificial means. It would be interesting to see if it can help restore the cells. Based on this perception, in previous experiments, we and some researchers artificially resumed the membrane sialic acids to levels similar to those found in young red blood cells (Dumaswala et al., 2000; Huang et al., 2016; Remigante et al., 2023b; Spinelli et al., 2023b; Stowell et al., 2013; Yang et al., 2006) and found that it was able to rejuvenate old erythrocytes and extend their lifespan. In this way, we successfully restored the youth of human erythrocytes aged approximately 90–120 days, as well as blood bank-stored human and rabbit erythrocytes, by incubating the aged RBCs with sialic acid (SA) and α-2,3-sialyltransferase. Remarkably, it was also able to recover some unhealthy cells (Huang et al., 2016). In addition to restoring cellular morphology, rheological properties, and overall viability, the structure and function of hemoglobin recovered nearly back to baseline levels typical of young normal cells. The revitalized cells demonstrated the ability to behave like young cells in circulation, as the survival time of the reinjected SA-restored erythrocytes was almost twice that of aged cells that had not undergone sialic acid remodeling. Routine blood examinations and health checks on the animals that received the reinjected SA-restored erythrocytes indicated that they were all in good condition, grew healthily, and exhibited typical behavior (Huang et al., 2016).

These facts demonstrated that using some artificial means to complement the deficient components of RBCs could retard the cells' aging process and even restore their youth. It also encourages us to hypothesize further that, besides resuming the surface SA, one may use some other methods to remodel/enhance the two vital proteins of RBCs (Hb and B3p) and protect them from the damage of oxidative stress, thereby delaying the aging process of the cells, or even rejuvenate the aged cells and recover the diseased ones.

Accordingly, in a preliminary study, we arranged a trial experiment to incubate RBCs with the solution of Hydroxysafflor yellow A (HSYA, a flavonoid). We found that the flavonoid could protect RBCs from acid-base imbalance, as the cells could keep their regular biconcave shape and membrane flexibility when their environmental pH value varied from 5.0 to 9.7. In contrast, the cells without HSYA action lost their natural shape and flexibility when pH was away from the neutral value. It indicated that the interference of some flavonoid-like substances, such as HSYA, can protect RBCs from oxidative injury, though the protection mechanism and the molecular action site, either Hb or B3p or both, were unclear yet.

Hydroxysafflor yellow A is a flavonoid extracted from safflower. Flavonoids have been shown to sequester reactive oxygen species (ROS) and enhance the activities of several antioxidant enzymes, including superoxide dismutase (SOD), catalase (CAT), and glutathione peroxidase (GPx). They also raise the glutathione (GSH) contents while reducing malondialdehyde (MDA) levels (Chagas et al., 2022).

Several studies have reported that various flavonoids and polyphenolic compounds, where flavonoids predominate, such as quercetin, chrysin, morin, Oleuropein, Açaì extract, blackcurrant fruit extract, and bergamot extract, have significant protective effects on erythrocytes against oxidative damage. For instance, when erythrocytes were exposed to quercetin and resveratrol under hypoxic conditions, the content of spectrin, ankyrin, B3p, and glyceraldehyde-3-phosphate dehydrogenase (GAPDH) of the cells increased compared to those in hypoxia without flavonoid treatment, even though they remained lower than in normoxic conditions. This effect helped maintain the regular discoidal shape of red blood cells even in hypoxia (Revin et al., 2019). Another flavonoid, the Açaì extract, demonstrated beneficial effects on band 3 phosphorylation and structural rearrangements of membrane cytoskeleton-associated proteins, such as α-β-spectrin, ankyrin, and protein 4.1, in RBCs treated with 100 mM D-Galactose (D-Gal). Pre-treatment with açaí berry extract mitigated the significant reduction in red blood cell membrane deformability caused by exposure to 100 mM D-Gal under hypoxic conditions (Spinelli et al., 2023a). Similar antioxidant and antihemolytic effects were observed in red blood cells pretreated with quercetin, fisetin, chrysin, morin, and 3-hydroxyflavone. The binding sites of these flavonoids were located near the ghost membrane to tryptophan residues of the membrane proteins, leading to an increase in membrane order and a partial arrest in the microviscosity of these membranes (Chaudhuri et al., 2007). Moreover, pre-incubation with quercetin significantly lowered glycated hemoglobin levels in erythrocytes treated with 100 mM D-Gal (Remigante et al., 2022). The oleuropein aglycone derivative 3,4-DHPEA-EDA could interact with red blood cell proteins to decrease membrane-bound hemoglobin and protect the cells from oxidative hemolysis induced by peroxyl radicals (Paiva-Martins et al., 2009). The bergamot peel and juice extract could prevent ROS production induced by D-Gal, restore alterations in the distribution and ion transport kinetics of band 3, inhibit over-activation of the endogenous antioxidant enzymes CAT and SOD, and prevent activation of glucose-6-phosphate dehydrogenase (G6PDH) (Remigante et al., 2023a).

All these studies indicated that flavonoids can enter red blood cells through passive diffusion. Approximately 90% of them bind to hemoglobin proteins, while a smaller portion is stored in the membrane compartment and binds to tryptophan residues of membrane proteins (Fiorani et al., 2003). On one hand, flavonoids had a significant anti-glycation effect on hemoglobin to increase the number of oxy-hemoglobin. On the other hand, they may act as regulatory fragments to alter the affinity between the anchoring protein and B3p by changing the phosphorylation state of B3p in hypoxic conditions. This alteration leads to an increased–SH capacity and a more ordered structure in the cell membrane (Fiorani et al., 2003). Thus, flavonoids are promising candidates for artificial interference aimed at simultaneously remodeling hemoglobin and B3p, which represent the potential molecular targets for therapeutic interventions.

While these findings are promising, the previous studies mainly focused on the protective effects of flavonoids on erythrocytes that were pretreated with various flavonoids and then incubated in a hypoxic environment induced by D-galactose or other oxidants, such as sodium nitroprusside and peroxyl radicals. The reported protective effects were also primarily related to oxidative damage, but did not address some types of damage that RBCs typically encounter during circulation or storage in blood banks. These include damage from mechanical stress, acid-base imbalances, and storage lesions. They did not study how long the protective effect would last. More importantly, these studies demonstrated only the protective effects of flavonoids on RBCs but did not indicate whether they can aid in the recovery of aged RBCs or those already oxidatively damaged. This latter concern is the central focus of the present manuscript.

Therefore, this study will focus on whether remodeling/enhancing the two proteins (Hb and B3p) with flavonoids can restore aged, senescent, and damaged/diseased RBCs to their youthful or normal state. We will also explore the possibility of using this means to prevent RBCs from aging and the damages that RBCs would encounter during circulation or storage in blood banks, including the mechanical injury induced by high-speed centrifugal washing and cell sorting, damages caused by acid-base imbalances, hypoxia, and storage lesions either due to storing in the blood bank condition or the storage solutions lack of energy sources, such as phosphate-buffered saline (PBS). Besides their effects on the cellular structure and functions, such as the size, morphology, deformability, and enzymatic activities, we would be mainly interested in how flavonoids affect intracellular hemoglobin proteins and the Band three proteins. We will examine the variations of their conformation, concentration, distribution, and oxygen-carrying capability before and after applying the flavonoids. The flavonoids used in the study were HSYA, Angelica sinensis (AS) extract, Astragalus membranaceus (AM) extract, and a compound of HSYA, AS, and Ligusticum wallichii (LW) extract (in a 1:1:1 ratio, abbreviated as CA). We selected these flavonoids because HSYA demonstrated excellent protective effects against damage from acid-base imbalances in our preliminary experiments, while AS, AM, and LW are traditional Chinese medicinal plants known for their effectiveness in replenishing and invigorating human blood.

## Materials and methods

### Erythrocytes

Four types of erythrocyte samples were prepared. They were:

*Human Red blood cells* from freshly drawn venous blood that were obtained from 12 young, healthy, non-smoking adult male volunteers (age 23–26 years, mostly laboratory personnel, providing written informed consent).

*Blood bank human Red blood cells*. The RBC units were from ten bags of leukoreduced CPDA-1 RBC units obtained from the Guangzhou Blood Services Center, China and stored under standard blood-bank conditions. The RBC samples were prepared from the RBC units stored for different time periods (3 days, 7 days, 14 days, or 21 days) and designated as Unit 3, Unit 7, Unit 14, and Unit 21, respectively.

*Diseased red blood cells* obtained from the venous blood collected from ten children of α and β-thalassemia (provided by Guang Zhou Women and Children’s Medical Center, with written informed consent). The detection judgement of the diseased cells was conducted by the Center using HPLC.

*Rabbit Red blood cells* prepared from freshly drawn venous blood collected from the auricular artery of 18 male rabbits (Japanese white rabbits).

Studies on blood of volunteers (providing informed written consent) and animals were approved by Ji Nan University Animal Care and Use Committee conforming to the Chinese Public Health Service Policy on Human Care and Use of Laboratory Animals.

The freshly drawn human and rabbit heparinized venous blood samples were centrifuged thrice at 2000 × g for 10 min at 4 °C with isotonic PBS (90 mM NaCl, 50 mM sodium phosphate, 5 mM KCl, 6 mM glucose, pH 7.4). Then, the washed RBCs were centrifuged at 2700 *g* for 20 min over Percoll gradients (Pharmacia, Sweden) as previously described (Huang et al., 2011a) to obtain four fractions of RBC. After centrifugation, the light cells (termed young or Y cells) and the dense cells (termed old or O cells) were harvested separately, washed twice in PBS, and stored at 4 °C until subsequent treatment and analyses (Tuo et al., 2014).

Similarly, for the blood bank stored units, 5 mL of the RBC suspension was taken from each RBC unit of various storing periods and randomly divided into five 1 mL parallel samples. They were also centrifuged at 2700 *g* for 20 min over Percoll gradients. Cells harvested from each fraction were washed twice in PBS and kept at 4 °C for subsequent treatment and analyses.

### Incubation of the RBC samples with flavonoids

Each parallel sample from various groups of the four types of erythrocytes was taken 50 μL to incubate with 50 μL HSYA (0.4 mg/ml, purchased from Shang Hai Yuan Ye Biotechnology Co.), 50 μL AS (100 mg/mL, purchased from Jiangxi Jiedu Pharmaceutical Co.), 50 μL AM (20 mg/mL, purchased from Hei Longjiang ZBD Pharmaceutical Co.), and 50 μL CA (100 mg/mL, purchased from Jiangxi Jiedu Pharmaceutical Co.), respectively, at 37 °C for 4 hours. They were then washed with 0.9% NaCl or PBS (1500r/min, each for 5min) for three times and suspended in different media (either 0.9% NaCl,or PBS,or PBS + BSA) for subsequent analyses.

### Creation of experimental hypoxia

The hypoxic environment was created using the AnaeroPack method (Kamiya et al., 1998). RBC samples incubated in Hepes solution with BSA were put in a sealed airtight container containing an AnaeroPack (Mitsubishi Gas Company, Tokyo, Japan) and an oxygen indicator. The container was placed in a CO_2_ incubator at 37 °C. The oxygen concentration in the container dropped to 0.1% in 1h and was maintained at this level during the experiment.

### Determination of morphological properties and cellular functions

All measurements were performed on five parallel samples in each group. Image analysis for morphological property measurements was performed on three images from each of the five samples, while that for Bessis classification was performed on a total of 450 images from the five samples, with the number of cells in each image frame ranging from tens to hundreds. The morphological properties, including size and the shape regular factor (REF), were measured using a PCO camera (Germany) built in a Nikon TE 300 inverted microscope with ×40 and ×60 objectives. The images of the cells were analyzed by the IPP microscopic image analyzing system to obtain the morphological properties of each one (Huang, 2005). The time-lapse imaging for bending modulus Kc and shear elasticity μ_c_ measurements was performed using our dynamic image analysis technique, as described previously (Li and Huang, 2006). The membrane deformability was also measured using a standard micropipette aspiration technique (Huang et al., 2016).

The Zeta potential of RBCs was determined using a Zeta PLUS potentiometric analyzer (Brookhaven Instrument Limited, USA) at 37 °C.

The Na^+^, K^+^-ATP and Ca^2+^, Mg^2+^-ATP enzymatic activities of erythrocyte were determined using the Elisa Kit purchased from Nanjing Jiancheng Bioengineering Institute. While the 2, 3-DPG content of erythrocyte was determined using a Elisa Kit bought from Wuhan EIAab Science Co.

### Determination of molecular conformation, distribution, and functions of Hb and B3p

The molecular conformation, distribution, and oxygen carrying functions of Hb were determined by Raman spectra measurement and line-mapping using a JY RAM INV confocal Raman system with a 514.2-nm excitation line from an Ar^+^ ion laser as described previously (Huang et al., 2016; Kang et al., 2008). While the conformation and content of B3p and the related cytoskeleton components were determined using SDS-PAGE in which samples were boiled for 10 min in the sample buffer containing tris-glycine at the ratio of 1:4. Electrophoresis was performed at a constant voltage of 120 V for 80 min. After electrophoresis, gels were stained with Coomassie blue.

### Determination of intracellular pH

The intracellular pH was determined by the method of BCECF-AM pHi measurement (Ozkan and Mutharasan, 2002) using the BCECF-AM fluorescent pH indicator purchased from Molecular Probes TM. The exciting lights were 439 nm and 490 nm, respectively.

### Data statistical analyses

All the parameters were measured from five parallel samples in each group, data were expressed as mean ± SD. Statistical analyses were performed with t-tests using SPSS 19.0 statistical software (IBM, Armonk, NY, USA) to determine whether or not there is a statistically significant difference between the means of each pair of groups. P-values were obtained by comparison of cells without or with the incorporation of each kind of flavonoid. ANOVA was also used for multiple group comparisons.

## Results

### Effect on old RBCs

**FIGURE 1:**
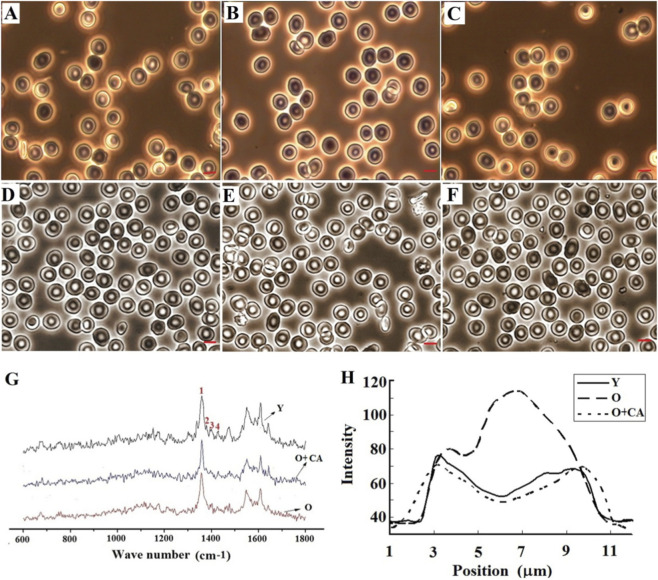
The images of young (Y), old (O), and O + CA human and rabbit RBCs and their Raman spectroscopy. **(A)** Y human RBCs, **(B)** O human RBCs, **(C)** O + CA human RBCs. **(D)** Y rabbit RBCs, **(E)** O rabbit RBCs, **(F)** O + CA rabbit RBCs, **(G)** The Raman spectra of Y, O, and O + CA RBCs, in which the spectra: 1) 1358 cm^-1^, 2) 1379 cm^-1^, 3) 1396 cm^-1^, 4) 1428 cm^-1^, **(H)** The line scanning Raman intensities indicating the Hb distribution across the cells.

Figures 1A–F show the morphologies of old human RBCs (hRBC) and rabbit RBCs (rRBC) obtained by Percoll separation and those incubated with HSYA and CA accompanied with that of young (Y) cells. The old (O) hRBCs obtained this way aged about 90–120 days in circulation, whereas Y hRBCs aged 1–30 days, as reported previously (Huang et al., 2011a). The detailed morphological parameters of the cells are presented in Table 1. We can see that the sizes of old cells were significantly smaller than Y cells. Some were not in regular biconcave discoid shape but with unsmooth periphery. However, after incubation with flavonoids, the old cells recovered their morphologies to almost the same as the young cells. The Zeta potential and bending modulus (*K*c) listed in the table indicated that the membrane surface charges and deformability of the old cells incubated with CA were restored to levels comparable to those of the young cells. From the table, we can also see that after incubating the old cells with CA, the activities of Na^+^, K^+^-ATPase, Ca^2+^, Mg^2+^-ATPase, and the level of 2,3-DPG nearly returned to the levels found in young cells. This recovery of viability parameters suggests that the two essential proteins, hemoglobin (Hb) and Band three protein (B3p), had recuperated their conformation and functionality as the young cells. Since the 2,3-DPG level in red blood cells reflects the affinity of hemoglobin for oxygen, its restoration indicates that the efficiency of oxygen unloading from hemoglobin has been restored. Additionally, membrane permeability is positively correlated with ATPase activities, and the activation of the ion pumps can significantly increase intracellular ATP levels. Thus, the restored ATPase activities suggested a recovery of the B3p conformation, leading to improved membrane permeability.

**TABLE 1:** Effect of flavonoids on the morphologies, Zeta potential, bending modulus (Kc), and viability parameters of human and rabbit RBCs.

Cells	N = 100	Contact area (μm^2^)	Long axis (μm)	REF
hRBC	Y	48.35 ± 6.18	8.0 ± 0.6	0.69 ± 0.07
​	O	41.17 ± 5.43 *	7.4 ± 0.6 **	0.73 ± 0.09 *
​	CA + O	48.39 ± 6.27	8.0 ± 0.6	0.68 ± 0.08
rRBC	Y	29.86 ± 2.81	6.3 ± 0.2	0.75 ± 0.05
​	O	27.65 ± 2.26 *	5.9 ± 0.3 **	0.81 ± 0.07 **
​	CA + O	30.01 ± 3.03	6.4 ± 0.4	0.73 ± 0.07

Parameters	Y hRBC	O hRBC	T hRBC	Y rRBC	O rRBC	T rRBC
ZETA (mv)	−32.84 ±0.94	−23.35** ±2.12	−32.46 ±2.23	−23.67 ±1.64	−17.81** ±1. 59	−22.89 ±3.63
Kc(10^−19^ J)	1.586 ±0.013	2.199** ±0.034	1.617 ±0.827	1,29 ±0.11	1,87* ±0.12	1,32 ±0.15
Cells		Y		O	O + CA	
Na^+^K^+^-ATPase (u/gHb)		26.871 ± 3.960		23.587 ± 4.717*	25.364 ± 4.381	
Ca^2+^Mg^2+^-ATPase (u/gHb)		9.612 ± 1.158		9.456 ± 1.646*	9.764 ± 1.422	
2,3-DPG (nmol/ml)		2.643 ± 0.212		2.121 ± 0.049*	2.440 ± 0.094	

The data are expressed as mean ± SD, *p < 0.05, ^**^ *P* < 0.01 (vs. young RBC).

REF: regular factor; T: treated with CA. Y: young RBC, O: old RBC, hRBC: human RBC, rRBC: rabbit RBC.

The Raman spectra (see Figure 1G) detected from the three types of cells also provided evidence demonstrating the conformation changes in the intracellular hemoglobin proteins of the cells. The O cells had a ∼4 cm^-1^ shift and lower peak intensity at the band of 1379 cm^-1^, which is attributed to the C-N stretching vibration of the protein. They also had a 3 cm^-1^ shift at 1396 cm^-1^ and 1428 cm^-1^, respectively, with lower peak intensities. The two bands correspond to ν (pyr quarter-ring) and ν (C_α_C_m_)_sym,_ respectively. Therefore, these changes indicated a deformation of the heme porphyrin ring in Hb. Moreover, the intensity increase of the characteristic band of heme at 1358 cm^-1^ shown in O cells revealed that the hemes in the hemoglobin proteins of O cells should be exposed. These conformation changes weakened the oxygen-carrying capacity of the proteins. However, upon incubation with CA, the Hb in the cells had similar band positions from 1358 cm^-1^ to 1428 cm^-1^ as Y cells, suggesting that the conformation of the proteins had almost returned to status as that of Y cells, thus leading to the recovery of the viability parameters mentioned above.

In addition to the changes in molecular conformation, the Raman line-scanning across the RBCs provided information not only on the distribution of Hb proteins in the cell but also on their aggregation status. The line scanning was performed using the 1358 cm^-1^ (ν4) band for Hb under 514.5 nm excitation light. This band was chosen because it has a strong signal and shows minimal variation under the measuring conditions, allowing us to detect variations in protein intensity with an accuracy of approximately 2.8% (Kang et al., 2008). Furthermore, the ν4 band offers insights into the electron population in the π* orbital. As shown in Figure 1H, the Hb intensity in O cells was significantly higher than in Y cells, particularly at the edges of the cells. This indicates both the aggregation of Hb molecules and their attachment to the cell membrane, as previously reported by Huang et al. (Huang et al., 2016). However, in the O cells that were incubated with CA, the distribution of Hb intensity was nearly identical to that of the Y cells. This suggests that the Hb proteins may have disaggregated and achieved a more uniform distribution within the cells due to the influence of flavonoid.

### Effect on aged RBCs stored in blood bank conditions

**FIGURE 2:**
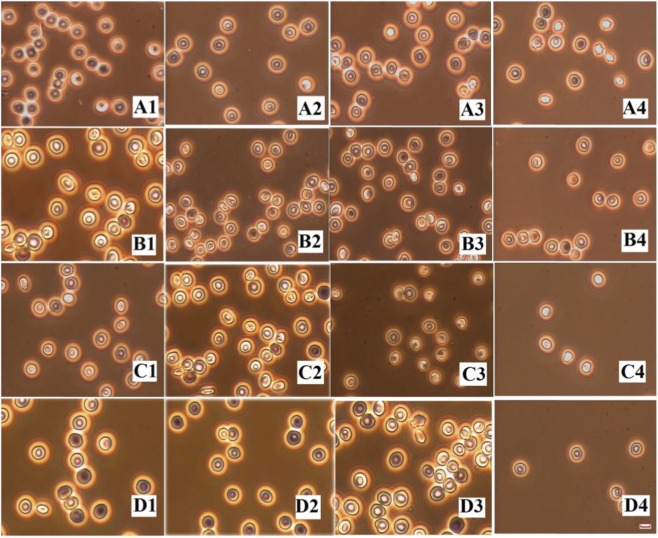
The images of the stored human RBCs. **(A1-A4)** The RBCs stored for 3, 7, 14, 21 days, respectively. **(B1-B4)** The RBCs treated with CA stored for 3, 7, 14, 21 days, respectively, **(C1-C4)** The RBCs treated with AM stored for 3,7,14,21 days, respectively, **(D1-D4)** The RBCs treated with HSYA stored for 3, 7, 14, 21 days, respectively.

The RBC samples stored for different periods (3 days, 7 days, 14 days, and 21 days) were incubated with CA, AM, and HSYA, respectively, to compare the effects of these flavonoids on the cells. Figure 2 shows the images of the four RBC units with and without incubating the three flavonoids. Table 2 provides detailed information on their morphological and biophysical properties and viability parameters. All the properties declined with storage time, and most were even lower than those of O RBCs from the freshly drawn blood (see Table 1), indicating that the RBCs stored in the blood bank aged faster than those in body circulation. The flavonoids could effectively restore all the properties of the cells, especially significant for those units stored for 14 and 21 days. Among them, CA was the most effective, followed by HSYA and then AM. The viability properties shown in Table 2 exhibited a similar trend, and the effectiveness of the flavonoids was again CA > HSYA>AM. An one-way ANOVA was also performed to compare the effects of CA, HSYA, and AM. It revealed that there were statistically significant differences in Contact area, Zeta potential, K_c_, Na^+^K^+^-ATPase,Ca^2+^Mg^2+^-ATPase, and DPG on the 14th and 21st day between CA and AM (p < 0.01); in Contact area, Zeta potential, K_c_ on third day and seventh day, Na^+^K^+^-ATPase, Ca^2+^Mg^2+^-ATPase, and DPG on the 14th and 21st day between HSYA and AM (p < 0.01); and in Contact area on the seventh day, Zeta potential on the 7th and 14th day between CA and HSYA (p < 0.01). There was no significant difference in Long axis and REF between CA and HSYA, between CA and AM, and between HSYA and AM (p > 0.05). Since CA showed the most significant effects on RBCs, we mainly used it to recover or protect the cells in the other experiments.

**TABLE 2:** Morphological, biophysical and viability parameters of stored RBCs with or without incubation with flavonoids.

Cells	Stored hRBCs				CA incubated stored hRBCs			
	3 days	7 days	14 days	21 days	3 days	7 days	14 days	21 days
Contact area/μm^2^	53.93 ± 4.62	45.64 ± 4.51	42.68 ± 5.00	43.14 ± 4.50	54.30 ± 5.07	48.50* ± 4.71	47.93** ± 4.30	45.53* ± 4.55
Long axis/μm	7.6 ± 0.5	7.4 ± 0.6	7.0 ± 0.6	7.0 ± 0.5	7.6 ± 0.6	7.4 ± 0.6	7.2 ± 0.6	6.9 ± 0.7
REF	0.64 ± 0.05	0.69 ± 0.06	0.74 ± 0.06	0.79 ± 0.06	0.81* ± 0.05	0.81* ± 0.06	0.83* ± 0.07	0.84* ± 0.05
ZETA (-mv)	28.05 ± 1.07	24.60 ± 1.00	21.97 ± 1.18	19.83 ± 1.13	29.31 ± 1.33	28.21* ± 1.02	31.58** ± 1.32	28.33** ± 1.22
*K*_c_(10^−19^ J)	2.116 ± 0.053	2.323 ± 0.055	2.672 ± 0.071	2.983 ± 0.126	2.107 ± 0.055	2.282 ± 0.054	2.536 ± 0.059	2.815 ± 0.068

Cells	AM incubated stored hRBCs				HSYA incubated stored hRBCs			
​	3 days	7 days	14 days	21 days	3 days	7 days	14 days	21 days
Contact area/μm^2^	47.88 ± 3.32	45.88 ± 4.23	43.83 ± 4.25	41.73 ± 4.61	53.90 ± 4.17	44.50 ± 4.32	46.83** ± 4.35	44.36* ± 4.56
Long axis/μm	7.3 ± 1.6	7.2 ± 0.9	7.1 ± 1.6	7.0 ± 0.9	7.5 ± 0.6	7.3 ± 0.6	7.1 ± 0.7	6.9 ± 0.7
REF	0.80* ± 0.05	0.87* ± 0.07	0.88* ± 0.06	0.89* ± 0.08	0.80* ± 0.08	0.82* ± 0.07	0.84* ± 0.06	0.83* ± 0.07
ZETA (-mv)	28.05 ± 1.27	24.78 ± 1.08	22.17 ± 1.17	20.90 ± 1.16	28.03 ± 1.46	26.32 ± 1.45	28.43* ± 1.65	28.02** ± 1.36
*K*_c_(10^−19^ J)	2.182 ± 0.064	2.353 ± 0.055	2.815 ± 0.063	2.932 ± 0.128	2.158 ± 0.065	2.312 ± 0.058	2.716 ± 0.063	2.822 ± 0.062

Cells	Stored hRBCs				CA incubated stored hRBCs			
​	3 days	7 days	14 days	21 days	3 days	7 days	14 days	21 days
Na^+^K^+^-ATPase (u/gHb)	25.364 ± 4.381	21.777 ± 2.939	11.078 ± 0.816	8.864 ± 0.570	27.004 ± 3.450	22.883 ± 3.558	11.970 ± 1.579	10.345* ± 0.810
Ca^2+^Mg^2+^-ATPase (u/gHb)	7.044 ± 0.482	6.538 ± 1.422	5.326 ± 0.962	4.327 ± 1.003	8.440 ± 0.948	7.657* ± 1.394	7.455** ± 1.158	6.936* ± 1.646
2,3-DPG (nmol/ml)	2.643 ± 0.212	1.996 ± 0.059	1.964 ± 0.034	1.839 ± 0.050	3.448* ± 0.075	3.278** ± 0.094	3.342** ± 0.111	3.439** ± 0.089

Cells	AM incubated stored hRBCs				HSYA incubated stored hRBCs			
​	3 days	7 days	14 days	21 days	3 days	7 days	14 days	21 days
Na^+^K^+^-ATPase (u/gHb)	25.587 ± 4.717	21.879 ± 1.571	11.626 ± 0.816	9.319 ± 0.405	26.871 ± 3.960	22.450 ± 2.985	11.848 ± 0.746	9.879 ± 0.573
Ca^2+^Mg^2+^-ATPase (u/gHb)	7.994 ± 0.462	6.632 ± 1.576	6.026 ± 0.678	5.745 ± 0.867	8.210 ± 0.868	7.076* ± 1.423	6.821* ± 1.236	6.212* ± 1.523
2,3-DPG (nmol/ml)	3.264 ± 0.266	3.296* ± 0.062	3.164** ± 0.036	3.183** ± 0.056	3.337* ± 0. 078	3.208** ± 0.089	3.246** ± 0.115	3.336** ± 0.121

^*^ *p* < 0.05, ^**^P < 0.01, vs. RBCs, without flavonoid treatment on the corresponding day.

### Effect on diseased RBCs

**FIGURE 3:**
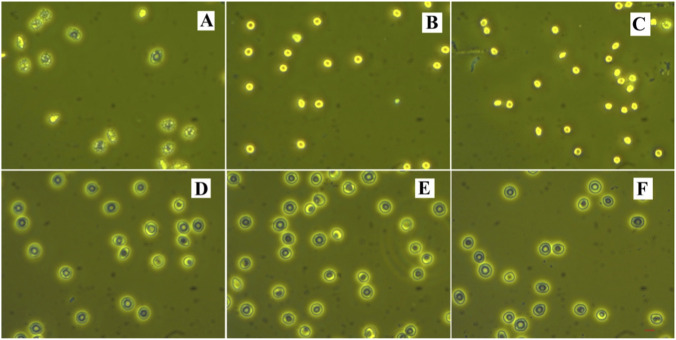
The images of diseased RBCs before and after treating with CA. **(A)** α-Thalassemia minor, **(B)** α-Thalassemia minima, **(C)** β-Thalassemia, **(D)** CA treated α-Thalassemia minor, **(E)** CA treated α-Thalassemia minima, **(F)** CA treated β-Thalassemia.

Figure 3 displays the images of α-Thalassemia minor, α-Thalassemia minimum, and β- Thalassemia RBCs before and after incubating with CA. All the diseased cells were in abnormal shapes and with smaller sizes than the normal ones: α-Thalassemia minor cells were echinocytes (III type), while α-Thalassemia minima and β-Thalassemia were stomatocytes (IV type). However, after incubating with CA, they were restored to biconcave discoid shape and almost ordinary sizes. Their Zeta potential, deformability, ATPases, and 2,3-DPG listed in Table 3 further proved the effectiveness of the flavonoid in recovering these diseased cells.

**TABLE 3:** The Zeta potential, deformability, and viability parameters of diseased RBCs with and without incubation with flavonoid.

​	Normal	α minor	α minor + CA	α minima	α minima + CA	β	β + AS
ZETA (mv)	−27.04 ± 0.94	−22.94 ± 1.47	−25.44* ± 5.13	−20.84 ± 3.64	−25.71** ± 2. 46	−22. 94 ± 1.47	−26.74* ± 2.23
Kc (10^−19^ J)	1.574 ± 0.536	4.328 ± 0.403	1.712** ± 0.015	​	​	4.328 ± 0.403	1.623 ± 0.325
Na^+^K^+^-ATPase (u/gHb)	25.364 ± 4.381	46.464 ± 5.638	27.264 ± 4.458	​	​	​	​
Ca^2+^Mg^2+^-ATPase (u/gHb)	7.994 ± 0.482	9.328 ± 1.232	8.023 ± 0.986	​	​	​	​
2,3-DPG (nmol/ml)	2.643 ± 0.212	3.623 ± 0.258	2.768 ± 0.236	​	​	​	​

*p < 0.05, ^**^ *P* < 0.01 (vs. the RBCs, without CA, treatment for each diseased group).

### Effect in protecting RBCs from mechanical injury

Washed red blood cells are often utilized to treat immune-related diseases and prevent allergic reactions in patients with severe responses to standard red blood cells. During RBC washing, the cells are centrifuged at 10,000 r/min (creating a centrifugal force of about 5000 × g) for 10 min. This process is typically repeated two to three times. During washing, the cells would experience mechanical injury, resulting in at least 20% loss of the cells, even when using freshly collected RBCs. However, incubating the RBCs with flavonoids before washing can help protect the cells from mechanical damage and reduce cell loss.

Table 4 illustrates the recovery rates of different RBC units with and without flavonoid incubation. For instance, for freshly collected RBCs (Unit 3), the recovery rate was 80.2% in the control group, while it increased to 83.4% for the RBCs incubated with CA before washing. The difference in recovery rates was even more pronounced for the units stored for more than 14 days. For example, the recovery rate of Unit 21, which underwent CA incubation, was 65.2%, significantly higher than the 44.3% recovery rate observed without flavonoid incubation. While HSYA showed less effectiveness in protecting the cells compared to CA, it still demonstrated a notable difference in recovery rates compared to the control groups.

**TABLE 4:** The recovery rate of RBCs after washing.

Unit	3 (days)	7 (days)	14 (days)	21 (days)	28 (days)
Control (%)	80.2 ± 2.1	77.9 ± 4.7	65.7 ± 2.8	44.3 ± 4.6	34.7 ± 5.8
CA + (%)	83.4 ± 3.5	82.3 ± 2.9	74.7 ± 1.9^*^	65.2 ± 2.4^**^	50.8 ± 3.7^*^
HSYA + (%)	​	78.2 ± 2.5	76.7 ± 2.7^*^	60.2 ± 3.1^**^	​

n = 3, ^*^p < 0.05, ^**^p < 0.01, vs. the control for each corresponding day.

### Effect in protecting RBCs from the storage lesions due to storing in solutions lack of energy sources (PBS)

**FIGURE 4:**
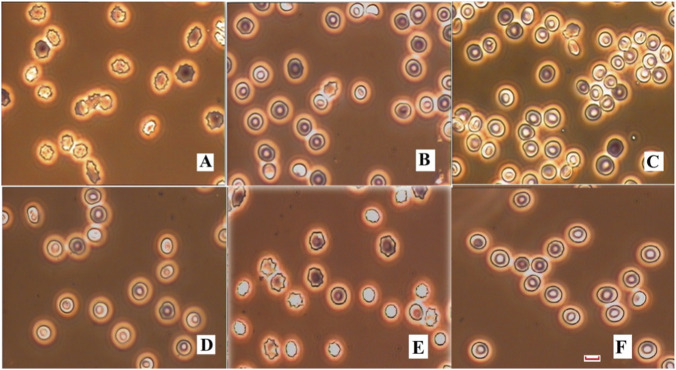
The images of RBCs stored in different solutions. **(A)** RBCs stored in PBS for 3 days, **(B)** RBCs stored in PBS + BSA for 3 days, **(C)** RBCs stored in PBS + CA for 3 days, **(D)** RBCs stored in PBS + AM for 3 days, **(E)** RBCs stored in PBS + BSA for 9 days, **(F)** RBCs stored in PBS + CA for 21 days.

Figure 4 shows the images of the RBCs (Unit 3) stored in PBS, PBS + BSA, and PBS + CA for 3 days, respectively. Supplementary Table 1 gives the detailed statistical results from 450 images of five parallel samples in each group according to the Bessis classification of RBCs. We can see that for the RBCs in the PBS solution, only 12% of the cells were regular discocytes. The others were echinocytes or spheroechinocytes. In contrast, in the PBS + CA solution, 92% of the cells were discocytes, a proportion higher than the 90% observed in the PBS + BSA solution. Notably, the RBCs suspended in PBS + HSYA also demonstrated a significantly higher percentage of cells (78%) in the regular biconcave discoid shape than the 12% in the PBS solution. It suggests again that the effect of HSYA was about 85% as effective as that of CA. A similar effect was observed in the RBCs suspended in the PBS + AM solution. After 9 days of suspension, due to the anaerobic glycolysis effect, the pH value of the PBS + BSA solution progressively declined from 7.11 to 6.93 (on the 21st day, it was even as low as 6.52). As a result, most RBCs in the solution had transformed into echinocytes or stomatocytes (Figure 4E). However, the cells in the PBS + CA solutions remained predominantly in the regular biconcave discoid shape (see Figure 4F) even up to 21 days. The parameters of Zeta potential, bending modulus (Kc), the activities of Na^+^, K^+^-ATPase, Ca^2+^, Mg^2+^-ATPase, and the level of 2,3-DPG listed in Supplementary Table 2 indicate that the PBS + CA solution was a better medium than PBS + BSA in maintaining the cells’ regular biochemical and biophysical properties for long-term storage.

### Effect in protecting RBCs from damage caused by acid-base imbalances

In this experiment, a 50 μL suspension of leuko-reduced red blood cells stored for 1 day was incubated with either CA or HSYA (50  μL at 100 mg/mL) at 37 °C for 4 h. Following this, the cells were washed thrice with saline (0.9% NaCl) or PBS at 1500 r/min for 5 min each time. The cells were then re-suspended in PBS + BSA solutions at various pH values (3.0, 4.0, 5.0, 6.7, 7.5, 8.5, 8.8, and 9.7). The control group was not incubated with flavonoids.

We have previously presented some images of the RBCs with and without incubation with HSYA in solutions of different pH values (Huang et al., 2018). Here, we only provide the Bessis classification of the RBCs in the control group and the CA+ and HSYA + groups at various pH levels (see Supplementary Tables 3-5). The statistical results were derived from 450 images from five parallel samples in each group. The results indicate that in the control group, most cells maintained a regular biconcave discoid shape only at pH 6.7 and 7.5. Around half of the cells became echinocytes or stomatocytes at pH 5.0 and 8.0, and none retained a regular discoid shape at pH 3.0, 4.0, 8.8, and 9.7.

**FIGURE 5:**
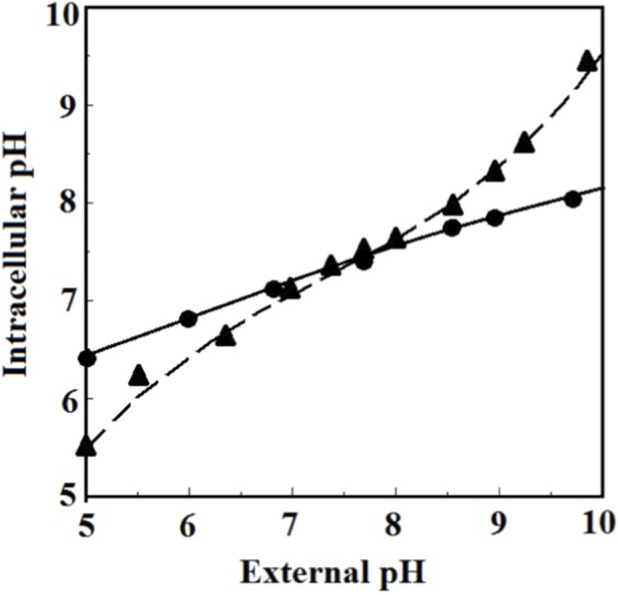
The intracellular pH values as functions of external pH. ●: CA pretreated RBC, ▲: Control.

In contrast, in the CA + group, more than 70% of the cells retained a regular biconcave discoid shape across pH values from 5.0 to 9.7. HSYA likewise demonstrated a protective effect on the cells, though it was less effective than CA. According to our previous study, HSYA could also maintain the normal deformability of the cells in a wide pH range (Huang et al., 2018). It indicates that flavonoids can effectively protect the cells from damage caused by acid-base imbalances, particularly alkaline damage. Figure 5 illustrates the intracellular pH as a function of the external pH for the cells with and without incubation with CA. It shows that CA could buffer the intracellular pH to change with the external one, so upon incubation with CA, the intracellular pH did not change abruptly with the external pH and remained within the range of 6.4–7.8 when the external pH varied from 5.0 to 9.7. It may explain why cells incubating with CA primarily retained their regular biconcave discoid shape in the pH range.

### Effect in protecting RBCs from the damage induced by hypoxia

**FIGURE 6:**
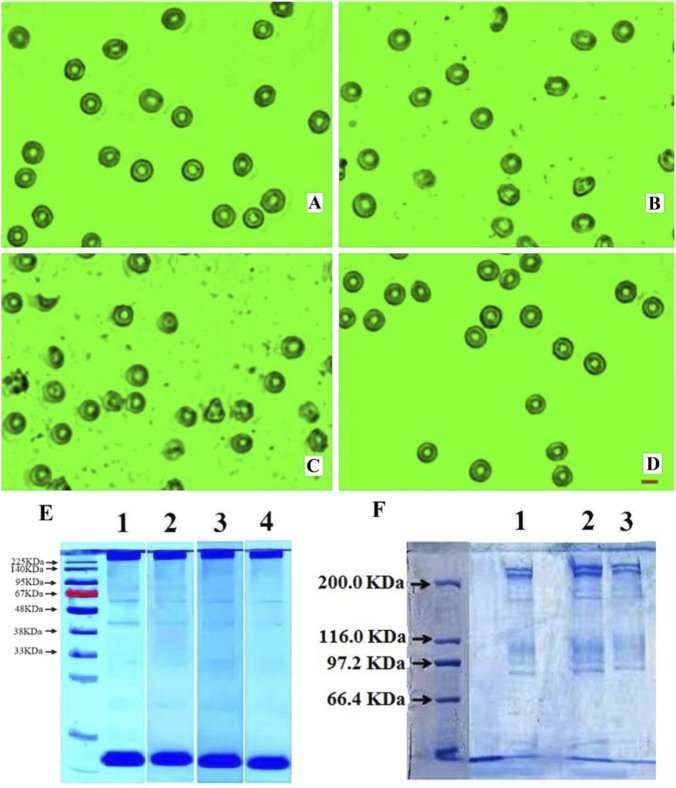
The images and SDS-PAGE of RBCs with and without hypoxic stress. **(A)** Normal RBCs without hypoxic stress, **(B)** RBCs in hypoxia for 17 h, **(C)** RBCs in hypoxia for 21 h, **(D)** RBCs with HSYA pretreatment in hypoxia for 21 h. **(E)** The SDS-PAGE of the RBCs with or without HSYA incubation in hypoxia, Group 1: normal control; Group 2: RBCs with 0.5 mg/mL HSYA pretreatment in hypoxia; Group 3: RBCs with 0.1 mg/mL HSYA pretreatment in hypoxia; Group 4: RBCs without HSYA pretreatment in hypoxia. **(F)** The B3p and cytoskeleton components of the RBCs with or without HSYA incubation in hypoxia, Group 1: RBCs without HSYA pretreatment in hypoxia; Group 2: RBCs with 0.3 mg/mL HSYA pretreatment in hypoxia; Group 3: RBCs with 0.1 mg/mL HSYA pretreatment in hypoxia.

Figures 6A–D present, respectively, the images of the RBCs in a PBS + BSA solution as the control, similar RBC suspensions placed in a hypoxic environment for 17 h and 21 h, and the RBCs pre-incubated with HSYA in the hypoxic environment for 21 h. The cells without pre-incubation with flavonoids in the hypoxic environment became echinocytes, and many micro-particles and membrane fragments appeared in the suspension, indicating that some cells had been disintegrated or underwent hemolysis. In contrast, the RBCs pre-incubated with HSYA could retain a regular biconcave discoid shape after being placed into the hypoxic environment for 21 h. The parameters listed in Supplementary Table 6 also indicate that HSYA could help the cells keep their size, surface charge, and deformability in the hypoxic environment similar to the control. However, the cells without incubating with the flavonoid lost their properties significantly in the hypoxic environment. Figure 6E illustrates the acrylamide gel electrophoresis (SDS-PAGE) of the RBCs with and without incubating HSYA under the effect of hypoxia. Figure 6F provides more detailed information on B3p and some other cytoskeleton components. They clearly show that the B3p (∼100 kDa) intensity of the HSYA + cells was similar to that of the control and significantly more intense than those without HSYA incubation, and the intensities of the groups of the RBCs with higher HSYA concentration were slightly more intense. The intensities of the other cytoskeleton components in the cell membrane, such as the band 1 protein (240 kDa) or spectrin (α chain), band 2 protein (220 kDa) or spectrin (β chain), band 2.1 protein (210 kDa) or ankyrin, band 4.1 (82 kDa), 4.2(76 kDa), and 4.5 (55 kDa), as well as band 5 protein (actin, 43 kDa), were also almost as intense as the control group. These facts suggest that after incubation with HSYA, the contents of B3p and the cytoskeleton components were conserved and did not undergo loss under hypoxia stress, indicating that HSYA could resist the damage to B3p, spectrin, ankyrin, and actin caused by hypoxic conditions.

## Discussion

We have demonstrated for the first time that using some artificial interference to remodel the two vital proteins (hemoglobin and B3p) of erythrocytes could rejuvenate aged or senescent cells and somewhat restore damaged or diseased ones to their normal state. We revealed that incubating the cells with HSYA-like flavonoids was one of the efficient means for the enhancement of the two proteins. This means could also prevent RBCs from aging and protect them against various types of harm, such as mechanical injury caused by high-speed centrifugal washing and sorting, damage caused by acid-base imbalances, storage lesions that occurred in blood bank conditions, or due to storage in energy-deficient storage solutions, and oxidative damage. Among the flavonoids we investigated, CA exhibited the highest efficiency in restoring and protecting red blood cells, while the pure single-component HSYA demonstrated approximately 80% of the effects observed with CA.

To understand why these flavonoids can restore and protect erythrocytes from aging and the damages caused by hypoxia, mechanical injury, storage lesions, acid-base imbalances, and diseases, first, we should review the general effects of flavonoids on erythrocytes. As previously described, flavonoids can enter erythrocytes through passive diffusion. After getting into the cells, most of them bind to hemoglobin proteins, while others accumulate in the membrane compartment and attach to tryptophan residues of membrane proteins. These interactions create an anti-glycation effect on hemoglobin and increase the amount of oxyhemoglobin. At the same time, flavonoids would increase–SH capacity and promote a more ordered structure in the cell membrane by enhancing the B3p phosphorylation and rearranging membrane cytoskeleton-associated proteins. Moreover, they decrease the levels of membrane-bound hemoglobin, restore the distribution and ion transport kinetics of B3p, prevent the production of reactive oxygen species (ROS), and enhance the endogenous antioxidant enzymes such as catalase (CAT) and superoxide dismutase (SOD). The negative charge density on the membrane surface also increases after the replacement of the structure of the erythrocyte membrane skeleton.

For the old RBCs aging in circulation, the cells become age accompanied by a series of changes in structure and functions, including the oxidation of Hb proteins, the formation of B3p clusters, and the loss of SA in circulation, etc. As demonstrated by the Raman spectra shown in Figure 1, when flavonoid got into the old cells and bound to Hb proteins, their anti-oxidation and anti-glycation properties helped restore the conformation of the Hb proteins. The Raman peaks of ν (pyr quarter-ring) and ν (C_α_C_m_)_sym,_ shifted back to 1396 cm^-1^ and 1428 cm^-1^, respectively, and the characteristic bands of heme also returned to 1358 cm^-1^-1428 cm^-1^ as that of Y cells, these facts indicated the conformation restoration. The Hb proteins disaggregated from aggregation and distributed uniformly in the cell again indicated by the line-scanning shown in Figure 1H also confirms the conformation restoration of the proteins due to the flavonoid effect. The restorations enabled the impaired proteins to regain their ability to bind oxygen, leading to an increased concentration of oxy-Hb as shown by the increased intensity of the spectra at 1396 cm^-1^ and 1428 cm^-1^ after the flavonoid treatment. Moreover, the restoration of 2,3-DPG levels following the addition of flavonoids also indicated an improved efficiency of oxygen unloading from hemoglobin. The recovered conformation of the Hb proteins prevented them from aggregating or adhering to the cell membrane, allowing them to distribute uniformly throughout the cell, similar to the distribution seen in young cells (refer to Figure 1F). At the same time, flavonoids functioned as regulatory agents that modify the affinity between the anchoring protein and B3p by changing the phosphorylation state of B3p. The cross-linking of B3p was thereby adjusted to facilitate the dissociation of B3p clusters that form during cell aging, leading to the restoration of B3p conformation. As a result, there was an increase in membrane order and an improvement in the distribution and ion transport kinetics of B3p. The recovered activities of Na^+^, K^+^-ATPase, and Ca^2+^, Mg^2+^-ATPase listed in Table 2 confirmed the effects. The restoration in the contents of B3p protein and the components of the cytoskeleton illustrated by the SDS-PAGE shown in Figures 6E,F, from another aspect, also demonstrated such a beneficial effect of flavonoids on B3p.

Once the two essential proteins, Hb and B3p, that were under degenerative change due to aging were remodeled to regain their proper conformation, concentration, distribution, and functionality, the viability properties of the cells recovered. The restored number and activity of RBC Na^+^-K^+^-ATPase enhanced the surface area-to-volume ratio and cytoplasmic rheology. The rebalancing of K^+^ and Na^+^ due to the recovered Na^+^-K^+^-ATPase activity prevented water loss from the cells and facilitated their reabsorption of water. Thereby, it resulted in the restoration of the cell size and morphology. Moreover, the recovery of membrane conformation led to the restoration of the deformability and the membrane’s surface charges of the cells.

The above-mentioned mechanism about the effect of flavonoids on old RBCs is also valid for explaining their influence on the aged RBCs stored in blood bank conditions, for the cells stored in the blood bank age in a similar way as the old cells in circulation, but at a faster rate. By comparing the data of Tables 1, 2, we can see that the cellular and molecular properties of the cells stored for 14 and 21 days in the blood bank were worse than those of old cells with 90–120 days of cellular age in body circulation and became senescent. It indicates that the current storage medium, such as the CPDA solution used for RBC storage in blood banks and our experiment, is inadequate. It may also explain why the transfusion of RBCs stored for 21 days can lead to adverse effects in recipients (Tuo et al., 2014). However, upon incubation with flavonoids, the stored cells could still effectively restore all their properties, from cell size, morphology, and zeta potential to bending modulus (or deformability). Some properties even improved to levels comparable to young RBCs. So it suggests that flavonoids can even rejuvenate senescent RBCs. It is interesting to note that the effectiveness of the flavonoids in recovering the senescent RBCs was CA > HSYA>AM. As described earlier, CA is a mixture of HSYA, AS, and LW. The main active ingredients of both AS and LW are Ligustilide and Ferulic acid (Chao and Lin, 2011; Liang et al., 2005). According to the result of our trial experiment (data not shown) on the two ingredients, neither Ligustilide nor Ferulic acid alone could restore aged RBCs from abnormal size and shape to normal-sized discocytes, as HSYA did. But both of them have a variety of beneficial effects on erythrocytes, including elevated CAT expression, reduced ROS production and Hb oxidation, improved Na^+^/K^+^-ATPase activity, and decreased phosphatidylserine exposure in erythrocyte oxidation (Chang et al., 2016; Chao and Lin, 2011; Ren et al., 2025). Therefore, understanding why Ligustilide or Ferulic acid alone was ineffective in recovering aged RBCs, while the combination of AS, LW, and HSYA produced greater effectiveness than HSYA alone, would be an interesting subject of future study. The fact also implies there might be some synergistic effects from the multi-component pharmacokinetics of the flavonoids. CA is an approved medication by China’s Ministry of Health for promoting blood circulation. It has been proven effective in treating various diseases correlated with blood circulation, but more research is needed to understand its underlying mechanisms. Our results may provide the cellular and molecular basis for understanding its effects on blood circulation.

Remarkably, CA demonstrates significant beneficial effects on three types of diseased red blood cells: α-thalassemia minor, α-thalassemia major, and β-thalassemia. It helps restore certain cellular properties, including size, shape, surface charge, membrane deformability, ATPase activity, and 2,3-DPG levels. After treatment with CA, the microcytic, hypochromic, and abnormally shaped red blood cells regained a biconcave discoid shape (see Figure 3). Their sizes (volumes) were also almost restored to those of healthy red blood cells (see Table 3). As noted earlier, the levels of ATPases and 2,3-DPG in erythrocytes reflect the conformation and functionality of two essential proteins: hemoglobin and B3p. The ATPase and 2,3-DPG parameters listed in Table 3 suggest that CA treatment has partially restored the diseased cells in nearly all cellular and molecular aspects due to its effects on the two proteins. These findings are promising and encourage further investigation into the mechanisms underlying these effects and their potential therapeutic applications. Thalassemia is an inherited blood disorder caused by mutations that affect the globin chain subunits of hemoglobin. It leads to inadequate hemoglobin production and the accumulation of insoluble unpaired chains. As a result, patients have significantly lower complete blood count (CBC) and mean corpuscular volume (MCV) values than normal. However, mature red blood cells (RBCs) have lost their nuclei and other cellular organelles, meaning that Thalassemia RBCs do not contain DNA or RNA to synthesize new abnormal proteins or influence the existing ones. Therefore, the cells treated with CA may have the potential to maintain their restored ordinary shape, size, and functions over time. Our experiment on the diseased RBCs was preliminary, but the results suggest that it is worthwhile to further investigate whether CA truly recovers Thalassemia cells in a more in-depth manner.

The protective effect of flavonoids on RBCs during high-speed washing was probably due to their ability to strengthen the B3 proteins in the cell membrane. The B3 protein is crucial for regulating various cellular functions and maintaining the structural integrity and stability of the cells. By altering the phosphorylation state of B3p, flavonoids enhance the interaction between the anchoring protein and B3p, resulting in a more organized cell membrane. This enhancement increased the deformability of the cells, allowing them to withstand better the mechanical stress caused by centrifugal force during the washing process, as shown in the data presented in Table 3. Washing RBCs for leukoreduced or washed red cells is common in blood banks worldwide. We hope our findings inspire blood banks to explore new strategies to reduce RBC loss during preparation.

Over the past few decades, various storage solutions have been developed to preserve donated RBCs in blood banks. One of the earliest and most basic solutions is phosphate-buffered saline (PBS). However, PBS lacks glucose and other energy sources, which provide limited nutrition to support RBC metabolism. Additionally, it has inadequate buffering capacity, which fails to prevent metabolic disturbances and acidosis during prolonged storage. As a result, only 12% of the RBCs stored in PBS maintained their regular biconcave discoid shape, while the others became echinocytes or spheroechinocytes. Bovine serum albumin (BSA) has a three-dimensional structure and function similar to human serum albumin and can catalyze various biochemical reactions in PBS. Therefore, adding BSA to PBS solution creates an environment that more closely resembles plasma, which helps RBCs resist biochemical and biomechanical storage damage. However, due to the acidification of the solution and the deterioration of BSA, RBCs experience accelerated aging in the PBS + BSA solution, allowing them to maintain their regular structure and function for less than 9 days, similar to those stored under regular blood bank conditions. Instead, the PBS + CA solution could preserve the cells' regular biochemical and biophysical properties for over 21 days, indicating that the flavonoid could prevent RBCs from aging during storage. This effect may be due to the ability of the flavonoids to elevate catalase (CAT) expression, decrease reactive oxygen species (ROS) production, reduce hemoglobin oxidation and denaturation, enhance B3p phosphorylation, improve ATPase activity, and increase 2,3-DPG levels in the cells. Moreover, CA can maintain the intracellular pH of RBCs during storage, as shown in Figure 5. CA could even rejuvenate aged and abnormal RBCs as described previously. Therefore, incorporating CA-like flavonoids into RBC storage solutions would be a promising strategy for developing an ideal long-term solution to prevent RBCs from accelerated aging caused by storage lesions.

When the pH value of a storage solution varied from 3.0 to 9.7, both Hb and B3p underwent conformational changes. At acidic pH, the oxidative injury of membranes was primarily associated with cytosolic hemoglobin. As more H^+^ ions entered the cells and bound to Hb proteins, the oxygenation configuration of these proteins changed. This change reduced the binding of O_2_ to Hb and caused more oxy-Hb to become met-Hb. The met-Hb proteins then tended to attach to the inner membrane of the cells, decreasing the membrane deformability. On the other hand, B3p played a significant role as an intracellular pH (pH_i_) sensor and transport effector. It detected changes in the intracellular bicarbonate concentration [HCO_3_^−^]_i_ through its intracellular anion binding site, allowing B3p to adjust HCO3^–^ transport to modulate acid influx or efflux, thus regulating the RBC pH_i_. As the cell’s external pH (pH_o_) changed, the cytoplasmic domain of B3p (cdB3) altered its conformation to decrease the protein’s binding to anchor proteins, increase its mobility, and promote the dissociation of B3p from anchor proteins, leading to their aggregation. At the same time, the membrane-spanning domain of B3p was also affected, which influenced its ion transport activity and could result in irregular acid efflux or influx. This disruption led to water loss and cell shrinkage. All these changes affected the size (volume), morphology, and membrane deformability of the cells and induced alterations in the concentration and distribution of cytosolic hemoglobin and the dissociation of tetrameric Hb into monomers or dimers (Wu et al., 2010).

For the RBCs pretreated with flavonoids, as aforementioned, most of the entered flavonoids bound to Hb proteins. Such a binding not only enhanced the anti-oxidation and anti-glycation ability of the proteins but also blocked them from binding with H^+^ to become Met-Hb anymore. Therefore, it prevented the proteins from aggregating and adhering to the cell membrane. At the same time, the other flavonoid that bound to B3p strengthened the structure and ion transportation kinetics of the proteins, thus retaining the membrane order, regulating the acid efflux and influx, and enhancing the mass transport of CO_2_ in the form of plasma HCO_3_ under pH_o_ variation. Besides these effects on the two vital proteins, the flavonoid also elevated the activity of the endogenous antioxidant enzyme CAT for more effective scavenging of the intracellular H_2_O_2_. All these then led to an effect of buffering the intracellular pH to change with the external one, as illustrated in Figure 5. Therefore, the pH_i_ could remain within the range of 6.4–7.8 when pH_o_ varied from 5.0 to 9.7, so the cells could keep their regular size, shape, and deformability in the pH range.

In the Introduction section, we described the anti-oxidative stress effect of flavonoid on erythrocytes found in previous studies. Similarly, HSYA implements its effort via the two vital proteins, B3p and Hb. Under oxidative stress, Hb would undergo glycosylation, and B3p would be phosphorylated. The glycosylation of Hb proteins not only converted them into met-Hbs but also impaired their oxygen-carrying capacity, leading to their aggregation or attachment to the inner membrane of the cells. On the other hand, the phosphorylation of B3p altered its interaction with the cytoskeleton, resulting in membrane destabilization. It also reduced their binding to anchor proteins and promoted them to be high polymers. Moreover, hypoxia caused the degradation of cytoskeleton proteins (such as bands 1, 2.1, 4.1, 4.2, and 5). These processes collectively reduced the size and volume of the cells, distorted their regular shape into echinocytes or spheroechinocytes, decreased the cell deformability, and might even lead to cell disintegration, creating microparticles and membrane fragments, as shown in Figures 6B,C. When cells were incubated with HSYA, the anti-glycation properties of HSYA helped increase the number of oxyhemoglobin and prevent Hb from aggregating or attaching to the membrane. Furthermore, HSYA inhibited the oxidation of free sulfhydryl groups and the glycosylation of B3p, thereby strengthening the protein’s structure and interaction with the cytoskeleton. This preservation of membrane integrity under oxidative stress also prevented the degradation of spectrin proteins. The significantly higher expression levels of B3p and the cytoskeleton proteins than those without HSYA treatment (see Figures 6E,F) provide evidence for these effects. The treated cells maintained similar size, shape, surface zeta potential, and deformability compared to the control group, as indicated in Figures 6A,D, demonstrating that the cells remained undamaged in the hypoxic environment even after 21 h. Moreover, the clean suspension of the treated RBCs (see Figure 6D) further confirms that the cells could retain their integrity in the hypoxic environment for 21 h, unlike the untreated cells, which exhibited numerous micro-particles and membrane fragments due to hemolysis (see Figure 6C). These facts suggest that the flavonoid could protect RBCs from severe hypoxia (only 0.1% oxygen concentration) for quite a long time, at least 21 h.

## Conclusion

We discovered that by remodeling both Hb and B3p, which undergo degenerative changes due to cell aging with certain flavonoids, such as HSYA, AS, AM, and CA, one could effectively rejuvenate aged or senescent RBCs. The flavonoid also had beneficial effects on damaged or diseased RBCs. Moreover, this method could protect RBCs from aging and various injuries, including mechanical injury caused by high-speed centrifugal washing and damage resulting from acid-base imbalances, storage lesions, and oxidative stress.

The results of Raman spectroscopy and SDS-PAGE demonstrated that during incubation with RBCs, flavonoids could bind to the two vital proteins Hb and B3p in RBCs and simultaneously remodel their protein conformation, content, concentration, distribution, and functions. This interaction leads to the recovery or enhancement of various cellular and molecular properties of the cells, including cell size, morphology, deformability, zeta potential, ATPase activity, 2,3-DPG levels, oxygen-carrying capability, and ion transportation activity.

The flavonoids could rejuvenate aged or recover some damaged/diseased RBCs in a couple of hours after incubating with the cells. Once inside the cells, these compounds can remain in the cells for an extended period in a biologically active form, offering protection against storage lesions and other damages for at least 21 days. They could also recover aged cells with storage lesions stored in blood bank conditions for more than 21 days, and the old cells with a cellular age of 90–120 days.

The primary focus of this manuscript is to demonstrate our hypothesis that utilizing artificial interference to remodel and enhance two critical proteins could rejuvenate aged or senescent cells and restore damaged red blood cells (RBCs). Therefore, we did not explore the pharmacological effects of flavonoids in-depth to determine optimal and minimal effective doses. Future studies are needed to examine their dose-dependent effects. Moreover, although the main active components of Angelica sinensis and Ligusticum wallichii were not found effective in recovering old RBCs and the RBCs aged in the blood bank condition, due to the synergistic effect of multi-component pharmacokinetics, the compound CA, which consisted of the two components and HSYA, was more efficient in recovering and protecting RBCs than the single active component HSYA. Therefore, besides the active components, one should also consider the synergism of multi-component pharmacokinetics in studying the beneficial effect of flavonoids on RBCs.

In summary, this study demonstrates that mammalian erythrocytes, which lack nuclei and other organelles, can have their aging delayed and lifespan extended through the remodeling and enhancement of two key proteins: hemoglobin (Hb) and protein B3p. Specifically, incubating these cells with flavonoids, such as hydroxysafflor yellow A (HSYA) and CA, can effectively induce this remodeling. This approach is advantageous because the flavonoids simultaneously target and remodel both Hb and B3p, resulting in the enhancement and restoration of a wide range of cellular and molecular properties. Compared to the sialic acid resumption methods we established previously (Huang et al., 2016), this new approach not only rejuvenates aged and recovers diseased red blood cells to some extent but also prevents various cellular damages. Additionally, the strategy of remodeling key proteins to enhance or rejuvenate cells is promising and could potentially be applied to other cell types. Therefore, it holds significant implications in multiple aspects of cellular and molecular biology and would have applications in various fields of biomedicine.

## Data Availability

The original contributions presented in the study are included in the article/Supplementary Material, further inquiries can be directed to the corresponding author.
