# Supplementary material for: Recover aged/damaged erythrocytes and prevent them from aging by remodeling or enhancing their vital proteins: Hb and B3p
Source: Front Cell Dev Biol. 2026 Apr 29;14:1781681. doi: 10.3389/fcell.2026.1781681 (PMC13182234; doi:10.3389/fcell.2026.1781681)
Supplement: Supplementary file 1 [file Table1.docx]

**Supplementary materials**

**Supp.Table 1 Statistical result of Bessis classification of RBCs stored in different solutions**

| Bessis分类 | PBS | PBS+BSA | PBS +CA | PBS +HSYA |
| --- | --- | --- | --- | --- |
| Discocyte | 12±3 % | 90±2 % | 92±1 % | 78±3% |
| Stomatocyte-b |  | 6±1 % |  | 10±2% |
| Stomatocyte-c |  |  | 5±1 % | 1±1% |
| Stomatocyte-d |  |  | 3±2 % |  |
| Spherostomatocyte |  |  |  |  |
| Echinocyte-f | 21±5 % | 4±2 % |  | 6±2% |
| Echinocyte-g | 7±4 % |  |  | 2±1% |
| Echinocyte-h | 25±3 % |  |  | 1±1% |
| Echinocyte-i | 27±6 % |  |  | 2±1% |
| Spheroechinocyte | 8±2 % |  |  |  |

**Supp. Table 2**. The biophysical properties and viability parameters of RBCs in PBS+BSA and PBS+CA solution

| Cells | RBCs in PBS+BSA solution | | | | RBCs in PBS+CA solution | | | |
| --- | --- | --- | --- | --- | --- | --- | --- | --- |
| Storage time | 3 days | 7 days | 14 days | 21 days | 3 days | 7 days | 14 days | 21 days |
| ZETA（-mv） | 28.01±1.12 | 23.47±  1.03 | 21.04±  1.23 | 19.95±  1.11 | 28.91±1.36 | 28.32*±  1.12 | 28.28**  ±1.25 | 27.36**  ±1.42 |
| *K*_c_(10^-19^ J) | 2.112±0.062 | 2.331±  0.053 | 2.676±  0.068 | 2.985±  0.096 | 2.106±0.045 | 2.278±  0.056 | 2.533±  0.062 | 2.802±  0.065 |
| Na^+^K^+^-ATPase（u/gHb） | 25.432±3.624 | 22.012±3.021 | 11.217  ±0.876 | 7.327  ±0.544 | 27.866±3.231 | 24.343  ±3.563 | 15.367*  ±1.673 | 11.542*  ±0.810 |
| Ca^2+^Mg^2+^-ATPase（u/gHb） | 7.152 ±0.398 | 6.572±  1.621 | 5.282±  1.263 | 4.289±  1.275 | 8.782±1.024 | 7.865*  ±1.256 | 7.721**  ±1.212 | 7.327*  ±1.536 |
| 2,3-DPG（nmol/ml） | 2.656±  0.189 | 1.993±  0.132 | 1.926±  0.125 | 1.834±  0.161 | 3.524* ±0. 066 | 3.352**  ±0.086 | 3.340**  ±0.122 | 3.338**  ±0.109 |

^*^p < 0.05, ^**^p < 0.01, compared with the one in PBS+BSA solution.

**Supp. Tables 3. Statistical result of Bessis Classification of control group at various pH values**

| Type pH value | 3.0 | 4.0 | 5.0 | 6.7 | 7.5 | 8.0 | 8.8 | 9.7 |
| --- | --- | --- | --- | --- | --- | --- | --- | --- |
| Discocyte | 0 | 0 | 58±2% | 82±3% | 87±2% | 46±4% | 0 | 0 |
| Stomatocyte | 0 | 29±2% | 5±1 % | 6±2 % | 0 | 0 | 0 | 0 |
| Spherostomatocyte | 100% | 71±3% | 0 | 0 | 0 | 0 | 0 | 0 |
| Echinocyte | 0 | 0 | 37±3% | 12±4% | 13±2% | 54±5% | 0 | 0 |
| Spheroechinocyte | 0 | 0 | 0 | 0 | 0 | 0 | 100% | 100% |

**Supp. Tables 4. Statistical result of Bessis Classification of CA+ groups at various pH values**

| Type pH value | 3.0 | 4.0 | 5.0 | 6.7 | 7.5 | 8.0 | 8.8 | 9.7 |
| --- | --- | --- | --- | --- | --- | --- | --- | --- |
| Discocyte | 0 | 0 | 74±3% | 88±4% | 91±3% | 88±2% | 83±1% | 80±1% |
| Stomatocyte | 2±1% | 17±3% | 26±4% | 12±2% | 9±2% | 12±1% | 17±2% | 15±3% |
| Spherostomatocyte | 98±2% | 83±2% | 0 | 0 | 0 | 0 | 0 | 0 |
| Echinocyte | 0 | 0 | 0 | 0 | 0 | 0 | 0 | 5% |
| Spheroechinocyte | 0 | 0 | 0 | 0 | 0 | 0 | 0 | 0 |

**Supp. Tables 5. Statistical result of Bessis Classification of HSYA+ groups at various pH values**

| Type pH value | 3.0 | 4.0 | 5.0 | 6.7 | 7.5 | 8.0 | 8.8 | 9.7 |
| --- | --- | --- | --- | --- | --- | --- | --- | --- |
| Discocyte | 0 | 0 | 59% | 79% | 88% | 68% | 61% | 15% |
| Stomatocyte | 0 | 20% | 2% | 3% | 3% | 0 | 0 | 0 |
| Spherostomatocyte | 6% | 79% | 0 | 0 | 0 | 0 | 0 | 0 |
| Echinocyte | 0 | 0 | 39% | 21% | 19% | 32% | 39% | 71% |
| Spheroechinocyte | 0 | 0 | 0 | 0 | 0 | 0 | 0 | 14% |
| Hemolysis | 94% | 1% | 0 | 0 | 0 | 0 | 0 | 0 |

**Supp. Table. 6**. The size and biophysical properties of RBCs in hypoxia environment with and without pretreating with HSYA

| Cells | Control | 17 h in hypoxic environment | 21 h in hypoxic  environment | HSYA+cells 21 h under hypoxia |
| --- | --- | --- | --- | --- |
| Contact area/μm^2^ | 53.93±4.62 | 44.64±3.68* | 42.68±4.23** | 53.14±3.52 |
| ZETA（-mv） | 28.05±1.07 | 26.80±1.21* | 22.69±1.32** | 27.83±1.18 |
| Kc(10^-19^ J) | 2.116±0.053 | 2.227±0.065 | 2.578±0.081* | 2.182±0.062 |

^*^p < 0.05, ^**^p < 0.01, compared with the control.
